# Comparison of the Formation of Plant–Microbial Interface in *Pisum sativum* L. and *Medicago truncatula* Gaertn. Nitrogen-Fixing Nodules

**DOI:** 10.3390/ijms241813850

**Published:** 2023-09-08

**Authors:** Anna V. Tsyganova, Elena V. Seliverstova, Viktor E. Tsyganov

**Affiliations:** 1 Laboratory of Molecular and Cell Biology, All-Russia Research Institute for Agricultural Microbiology, Saint Petersburg 196608, Russia; elena306@yandex.ru (E.V.S.); vetsyganov@arriam.ru (V.E.T.); 2 Sechenov Institute of Evolutionary Physiology and Biochemistry of the Russian Academy of Sciences, Saint Petersburg 194223, Russia

**Keywords:** cell wall, pectin, xyloglucan, arabinogalactan protein, extensin, infection thread, root nodule

## Abstract

Different components of the symbiotic interface play an important role in providing positional information during rhizobial infection and nodule development: successive changes in cell morphology correspond to subsequent changes in the molecular architecture of the apoplast and the associated surface structures. The localisation and distribution of pectins, xyloglucans, and cell wall proteins in symbiotic nodules of *Pisum sativum* and *Medicago truncatula* were studied using immunofluorescence and immunogold analysis in wild-type and ineffective mutant nodules. As a result, the ontogenetic changes in the symbiotic interface in the nodules of both species were described. Some differences in the patterns of distribution of cell wall polysaccharides and proteins between wild-type and mutant nodules can be explained by the activation of defence reaction or premature senescence in mutants. The absence of fucosylated xyloglucan in the cell walls in the *P. sativum* nodules, as well as its predominant accumulation in the cell walls of uninfected cells in the *M. truncatula* nodules, and the presence of the rhamnogalacturonan I (unbranched) backbone in meristematic cells in *P. sativum* can be attributed to the most striking species-specific features of the symbiotic interface.

## 1. Introduction

In the course of evolution, plants used certain functional capabilities of microorganisms to expand their adaptive potential. Thus, legumes acquired the ability to develop endosymbiotic relationships with proteobacteria called rhizobia that reduce atmospheric nitrogen to ammonia [1,2]. Symbiotic bacteria fix nitrogen in a reaction catalysed by the enzyme nitrogenase and provide it to plants in exchange for carbon sources. One of the features associated with symbiotic interaction is the formation of specialised organs called nodules, which create a favourable microenvironment for bacterial nitrogenase activity [3]. Nodule development is provided by two parallel but interrelated processes: nodule organogenesis and its infection (colonisation) with rhizobia [4]. The accommodation of bacteria within plant cells is one of the most unique features of this mutualistic association.

Cell walls and plasma membranes are involved in the exchange of substances between partners during the interaction of plants and microorganisms, while symbiotic interactions achieve full functionality due to the development of an extensive contact surface between the host and the microsymbiont—the symbiotic interface [5,6]. Despite the identification of components of the signalling cascade that initiates infection and the transcriptional network responsible for host cell reprogramming [7,8,9], analysis of complex cell wall components as polysaccharides and structural proteins is difficult due to the structural complexity of these macromolecules. Therefore, along with molecular methods, the use of monoclonal antibodies that react with the components of the symbiotic interface, including plant and bacterial cell wall polysaccharides, glycoproteins, and glycolipids, is an appropriate approach for studying these macromolecules [10,11,12,13,14,15,16].

Different components of the symbiotic interface play an important role in providing positional information during rhizobial infection: successive changes in cell morphology correspond to sequential changes in the molecular architecture of the apoplast and the associated surface structures [5,17,18,19,20]. The extracellular matrix, including cell wall glycoproteins and pectins, is modified to ensure the development of infection threads and infected cells [21]. The subsequent release of rhizobia depends on the surface properties of the plasma membrane, which includes membrane arabinogalactan proteins and glycolipids [22,23]. During the differentiation of symbiosomes and their transition to nitrogen fixation, the directions of vesicular transport are modified to control bacteroid metabolism. In turn, the functioning of symbiosomes is interrupted after their senescence during the autolytic process. In addition, the process of colonisation largely depends on the suppression of host defence reactions, both in the extracellular matrix and in the cytoplasmic phase of the existence of rhizobia [24,25,26].

In recent decades, various “omics” studies have made significant progress in the identification of a number of important molecular players in legume-rhizobial symbiosis [27,28,29,30,31]. An additional toolkit was formed after obtaining numerous mutants interrupted at various stages of nodule development [32,33]. At the same time, the processes occurring in the cell wall, one of the most important compartments of the plant cell and the key component of the symbiotic interface, remain poorly characterised, which is confirmed by the description of significant and unusual modifications of various symbiotic structures during nodule formation and functioning [12,34,35]. The lack of detailed information about the processes in the cell wall that occur during the interaction with rhizobia is explained by the complexity of the organisation of this multicomponent structure and the associated difficulties in its characterisation, as well as the remaining underestimation of the cell wall as a compartment that performs numerous functions and directly dictates many events of morphogenesis and signalling in plants. The diversity of the component composition of the apoplast, not only of the cell wall but also of the intercellular substance, where the host–plant interaction occurs, helps to explain one of the main phenomena of rhizobia diversity—host specificity.

Thus, the symbiotic interface, being a combination of both bacterial and plant factors, is responsible for the effectiveness of the interaction and serves as an indisputable specialised component of an effective symbiotic association. However, the development and modification of the symbiotic interface in legume nodules is still poorly understood.

In the present study, we obtained new data on the localisation and distribution of pectins, fucosylated xyloglucan, and cell wall proteins in nitrogen-fixing nodules of *Pisum sativum* and *Medicago truncatula*. Since the nodules of these legumes are of an indeterminate type, they can trace the gradient of cell differentiation when they are infected with rhizobia. Thus, this made it possible to consider the ontogenetic changes in the symbiotic interface. At the initial stages of infection, fucosylated xyloglucan, homogalacturonan molecules cross-linked by Ca^2+^ ions, and arabinan side chains of rhamnogalacturonan I molecules appear in cell walls. With further development of the infection, fucosylated xyloglucan disappears from the cell walls, but the content of homogalacturonan with a low degree of methylation increases, which reaches a maximum in senescent infected cells with degenerating symbiosomes. When bacteria release from infection threads (juvenile symbiosomes), arabinans similar to arabinan side chains of rhamnogalacturonan I are common in symbiosomes, but when symbiosomes mature and bacteroids differentiate, arabinan epitopes disappear.

## 2. Results

### 2.1. Methylesterified Pectin

**Figure 1:**
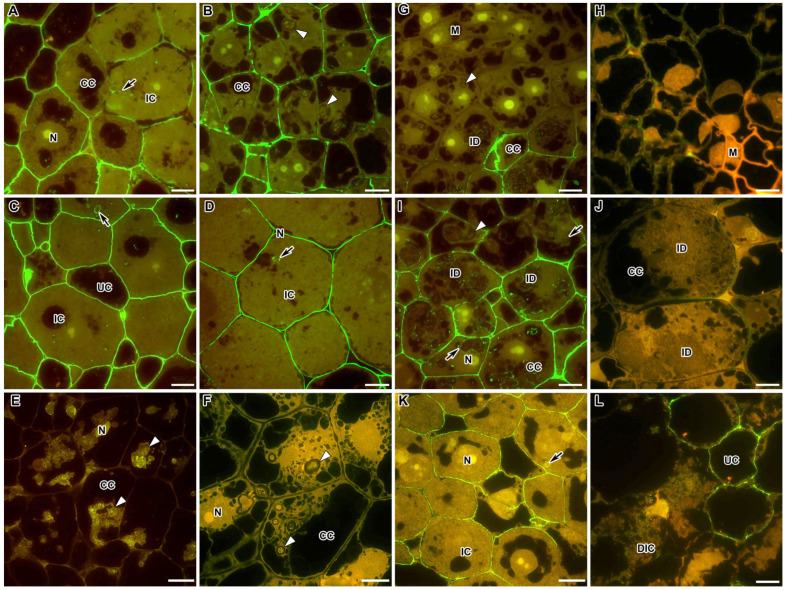
Fluorescent immunolocalisation of highly methylesterified homogalacturonan epitope labelled with LM20 in nodules from wild-type and mutant *Medicago truncatula* (**A**,**C**,**E**,**G**,**I**) and *Pisum sativum* (**B**,**D**,**F**,**H**,**J**–**L**). The secondary antibody used was the goat anti-rat IgG monoclonal antibody conjugated with Alexa Fluor 488. (**A**) Infection zone of the wild-type A17; (**B**) infection zone of the wild-type SGE; (**C**) nitrogen fixation zone of the wild-type A17; (**D**) nitrogen fixation zone of the wild-type SGE; (**E**) colonised cells of the mutant TR3 (*ipd3*); (**F**) colonised cells of the mutant SGEFix^−^-2 (*sym33*); (**G**) meristem of the mutant *efd–1*; (**H**) meristem of the mutant SGEFix^−^-1 (*sym40*); (**I**) infection zone of the mutant *efd–1*; (**J**) infection zone of the mutant SGEFix^−^-1 (*sym40*); (**K**) zone corresponding to nitrogen fixation zone of the wild type in the mutant SGEFix^−^-3 (*sym26*); (**L**) senescence zone of the mutant SGEFix^−^-3 (*sym26*). M—meristematic cell, CC—colonised cell, IC—infected cell, UC—uninfected cell, DIC—degraded infected cell, N—nucleus, ID—infection droplet; arrows indicate infection threads, triangles indicate infection threads without signal. Bar = 10 μm.

In the nodules of *M. truncatula* and *P. sativum* in the infection zone, highly methylesterified homogalacturonan (HG) accumulated mainly in the plant cell walls, and a reduced amount was observed in the infection thread walls (Figure 1A,B). At the same time, in the nodules of *P. sativum*, the accumulation of highly methylesterified HG in the cell walls was intermittent (Figure 1B). In the nitrogen fixation zone in both legumes, a homogeneous accumulation of the LM20 label was observed in the cell walls and the infection thread walls (Figure 1C,D). The LM20 label in the cell walls and infection thread walls was confirmed via electron microscopy (Figure S1A,B).

In the mutants of *M. truncatula* TR3 (*ipd3*) and *P. sativum* SGEFix^–^-2 (*sym33-3*) for the orthologous genes, the LM20 label was practically absent in the cell walls and infection thread walls (Figure 1E,F), which can serve as an indicator of the strengthening of the cell walls of these mutants, in the nodules of which there is no release of bacteria.

In the *M. truncatula* mutant *efd–1*, the epitope of highly methylesterified HG, labelled with the LM20 antibody, was absent in the meristem and early infection zone (Figure 1G); furthermore, it was localised not only in the cell walls and infection thread walls but also in the form of dots in infection droplets (Figure 1I). In the *P. sativum* mutant SGEFix^–^-1 (*sym40-1*) orthologous for the gene *EFD*, the LM20 label of HG accumulated in nodules in the area of the three-cell junctions in the meristem and early infection zone (Figure 1H), and traces of this epitope were found in the cell walls (Figure 1J).

In nodules of the *P. sativum* mutant SGEFix^–^-3 (*sym26*), characterised by early senescence, similarly to the wild-type nodules, the LM20 label of highly methylesterified HG was observed in the cell walls and infection thread walls in the zone corresponding to the nitrogen fixation zone of the wild-type nodules (Figure 1K). However, during senescence, this epitope disappeared from the cell walls of degrading infected cells, while remaining in the cell walls of uninfected cells (Figure 1L).

### 2.2. Demethylesterified Pectin

**Figure 2:**
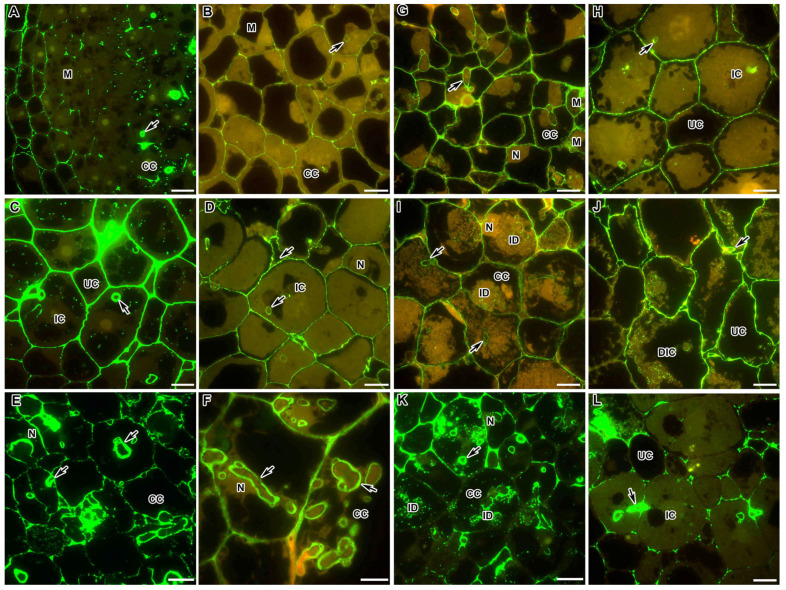
Fluorescent immunolocalisation of the low methylesterified homogalacturonan epitope labelled with LM19 in nodules from wild-type and mutant *Medicago truncatula* (**A**,**C**,**E**,**K**,**L**) and *Pisum sativum* (**B**,**D**,**F**–**J**). The secondary antibody used was the goat anti-rat IgG monoclonal antibody conjugated with Alexa Fluor 488. (**A**) Meristem and infection zone of the wild-type A17; (**B**) meristem and infection zone of the wild-type SGE; (**C**) nitrogen fixation zone of the wild-type A17; (**D**) nitrogen fixation zone of the wild-type SGE; (**E**) colonised cells of the mutant TR3 (*ipd3*); (**F**) colonised cells of the mutant SGEFix^−^-2 (*sym33-3*); (**G**) meristem and early infection zone of the mutant SGEFix^−^-1 (*sym40-1*); (**H**) zone corresponding to the nitrogen fixation zone of the wild type in the mutant SGEFix^−^-3 (*sym26*); (**I**) infection zone of the mutant SGEFix^−^-1 (*sym40-1*); (**J**) senescence zone of the mutant SGEFix^−^-3 (*sym26*); (**K**) infection zone of the mutant *efd–1*; (**L**) infection zone of the mutant *dnf1–1*. M—meristematic cell, CC—colonised cell, IC—infected cell, UC—uninfected cell, DIC—degraded infected cell, N—nucleus, ID—infection droplet; arrows indicate infection threads. Bar = 10 μm.

In the wild-type nodules of *M. truncatula* in the meristem and the infection zone, de-esterified HG was localised only in the infection thread walls and at the three-cell junctions (Figure 2A). On the contrary, in *P. sativum* nodules, this epitope was found in all cell walls in the meristem and the infection zone (Figure 2B). In the nitrogen fixation zone in the nodules of both species, the accumulation of de-esterified HG in cell walls increased (Figure 2C,D). Immunogold analysis confirmed the localisation of the LM19 epitope at the three-cell junctions (Figure S1C) and abundant accumulation in the infection thread walls in *M. truncatula* nodules (Figure S1D).

In the *M. truncatula* mutant TR3 (*ipd3*), which is characterised by the absence of bacterial release from the infection threads, the level of LM19 labelling was stronger in the infection thread walls than in the cell walls (Figure 2E). In the *P. sativum* mutant SGEFix^–^-2 (*sym33-3*), de-esterified HG, visualised with the LM19 antibody, was equally abundant in both the thickened cell walls and thickened infection thread walls (Figure 2F).

In the *P. sativum* mutant SGEFix^–^-1 (*sym40-1*), which is characterised by hypertrophied infection droplets, the LM19 label was more abundant in the meristem and early infection zone compared to the wild type (Figure 2G). In cells with hypertrophied infection droplets, the label of de-esterified HG in the cell walls and infection thread walls became less abundant, but appeared in the infection droplets as separate dots (Figure 2I). In the *M. truncatula* mutant *efd–1*, demethylesterified HG was localised mainly in the walls of infection threads, at the three-cell junctions, as dots in hypertrophied infection droplets, and in the walls of some infected cells (Figure 2K).

In the *M. truncatula* mutant *dnf1-1* with undifferentiated bacteroids, the pattern of distribution of de-esterified HG corresponded to the pattern of distribution of this epitope in the infection zone in the wild-type nodules (Figure 2L). In the *P. sativum* mutant SGEFix^–^-3 (*sym26*), which is characterised by early senescence, in the zone corresponding to the wild-type nitrogen fixation zone, the localisation and intensity of the LM19 label corresponded to those in the wild type (Figure 2H); in the senescence zone in the walls of degrading infected cells, the label became more abundant (Figure 2J).

### 2.3. Pectin Cross-Linked by Ca^2+^ Ions

**Figure 3:**
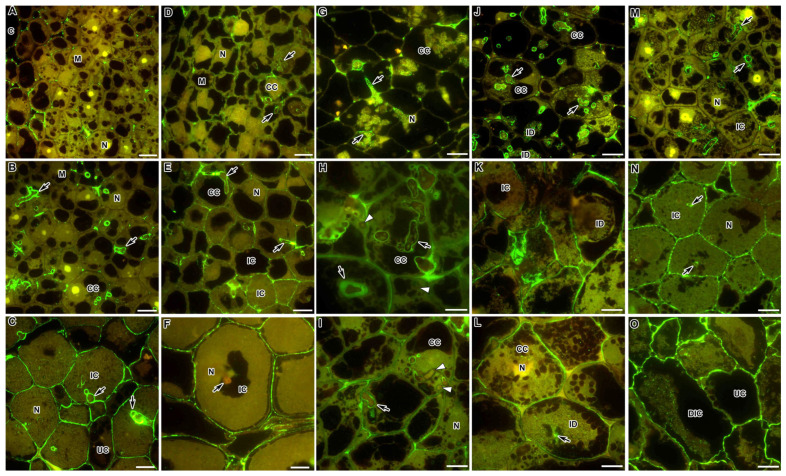
Fluorescent immunolocalisation of the dimeric association of homogalacturonan chains through Ca^2+^ ions labelled with 2F4 in nodules from wild-type and mutant *Medicago truncatula* (**A**–**C**,**G**,**J**,**M**) and *Pisum sativum* (**D**–**F**,**H**,**I**,**K**,**L**,**N**,**O**). The secondary antibody used was the goat anti-mouse IgG monoclonal antibody conjugated with Alexa Fluor 488. (**A**) Meristem of the wild-type A17; (**B**) infection zone of the wild-type A17; (**C**) nitrogen fixation zone of the wild-type A17; (**D**) meristem of the wild-type SGE; (**E**) infection zone of the wild-type SGE; (**F**) nitrogen fixation zone of the wild-type SGE; (**G**) colonised cells of the mutant TR3 (*ipd3*); (**H**,**I**) colonised cells of the mutant SGEFix^−^-2 (*sym33-3*); (**J**) infection zone in the mutant *efd–1*; (**K**) early infection zone of the mutant SGEFix^−^-1 (*sym40-1*); (**L**) late infection zone of the mutant SGEFix^−^-1 (*sym40-1*); (**M**) infection zone in the mutant *dnf1–1*; (**N**) zone corresponding to the nitrogen fixation zone of the wild type in the mutant SGEFix^−^-3 (*sym26*); (**O**) senescence zone of the mutant SGEFix^−^-3 (*sym26*). M—meristematic cell, CC—colonised cell, IC—infected cell, UC—uninfected cell, DIC—degraded infected cell, N—nucleus, ID—infection droplet; arrows indicate infection threads, triangles indicate infection thread without signal. Bar = 10 μm.

In the wild-type nodules of both *M. truncatula* and *P. sativum*, dimeric association of HG chains through Ca^2+^ ions, recognised by the 2F4 antibody, in the meristem (Figure 3A,D, respectively) and the infection zone (Figure 3B,E, respectively) accumulated mainly in the infection thread walls and at the three-cell junctions; in the nitrogen fixation zone, this epitope was evenly distributed in the cell walls (Figure 3C,F, respectively). However, in *P. sativum* nodules, in contrast to *M. truncatula*, this epitope was found in the cell walls in the infection zone (Figure 3E), while in the nitrogen fixation zone, it was not observed in the infection thread walls (Figure 3F). Immunogold analysis confirmed the localisation of the 2F4 epitope at the three-cell junctions and the abundant accumulation in the infection thread walls (Figure S1E,F).

In all *M. truncatula* mutants, the localisation of the calcium-bound HG epitope, recognised by the 2F4 antibody, basically coincided with the localisation in the nodules of the wild type observed in the infection zone, i.e., in the infection thread walls and at the three-cell junctions (Figure 3G,J,M). At the same time, in the mutant *efd-1*, the label was sometimes observed in the form of dots in infection droplets (Figure 3J), and in the mutant TR3 (*ipd3*), excessive accumulation of HG bound by Ca^2+^ ions was observed in the intercellular space (Figure 3G).

In the *P. sativum* mutants SGEFix^–^-2 (*sym33-3*) and SGEFix^–^-1 (*sym40-1*), the epitope of HG bound by calcium ions was localised in the cell walls and the infection thread walls in colonised cells in the infection zone, respectively (Figure 3H,I,K,L). However, in some infection threads in the mutant SGEFix^–^-2 (*sym33-3*), labelling with the 2F4 was weak or absent (Figure 3H,I). In the early senescent mutant SGEFix^–^-3 (*sym26*), the same pattern of the 2F4 epitope localisation was observed in infected cells as in the wild type (Figure 3N), and with the degradation of infected cells, the signal was increased (Figure 3O).

### 2.4. Rhamnogalacturonan I (Unbranched) Backbone

**Figure 4:**
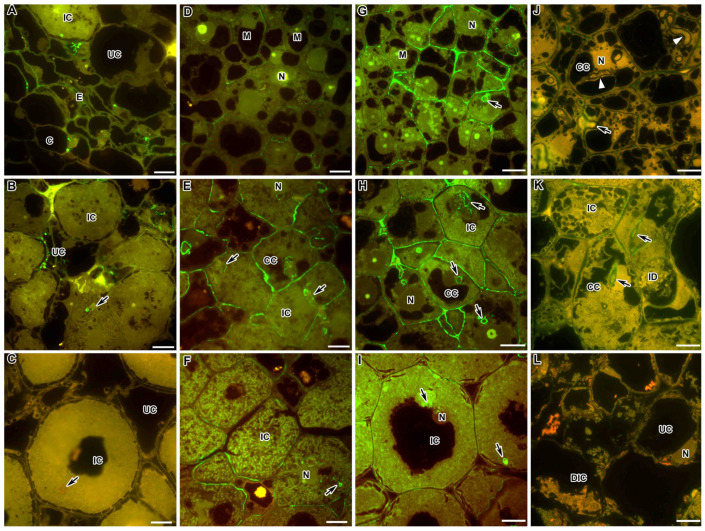
Fluorescent immunolocalisation of the rhamnogalacturonan I backbone labelled with CCRC-M35 (**A**–**C**) and CCRC-M36 (**D**–**L**) in nodules from wild-type and mutant *Medicago truncatula* (**A**,**B**,**D**–**F**) and *Pisum sativum* (**C**,**G**–**L**). The secondary antibody used was the goat anti-mouse IgG monoclonal antibody conjugated with Alexa Fluor 488. (**A**) Endodermis of the wild-type A17; (**B**) nitrogen fixation zone of the wild-type A17; (**C**) nitrogen fixation zone of the wild-type SGE; (**D**) meristem of the wild-type A17; (**E**) infection zone of the wild-type A17; (**F**) nitrogen fixation zone of the wild-type A17; (**G**) meristem of the wild-type SGE; (**H**) infection zone of the wild-type SGE; (**I**) nitrogen fixation zone of the wild-type SGE; (**J**) colonised cells of the mutant SGEFix^−^-2 (*sym33-3*); (**K**) infection zone in the mutant SGEFix^−^-1 (*sym40-1*); (**L**) senescence zone of the mutant SGEFix^−^-3 (*sym26*). C—cortex, E—endodermis, M—meristematic cell, CC—colonised cell, IC—infected cell, UC—uninfected cell, DIC—degraded infected cell, N—nucleus, ID—infection droplet; arrows indicate infection threads. Bar = 10 μm.

The following antibodies have been used to identify and localise rhamnogalacturonan I (RG-I): CCRC-M35, which binds to the RG-I backbone, requires at least two unbranched disaccharide repeats for binding, does not bind to branched sections of the backbone and is not sensitive to sugar identity at the non-reducing end; CCRC-M36, which requires at least three unbranched disaccharide repeats for binding, does not bind to branched sections of the backbone and is sensitive to sugar identity at the non-reducing end, preferring Rha over GalA. Immunocytochemical analysis of RG-I epitopes to the polysaccharide backbone of the molecule, labelled with CCRC-M35 and CCRC-M36 antibodies, showed the presence of RG-I molecules in the wild-type nodules of both *M. truncatula* (Figure 4A,B,D–F) and *P. sativum* (Figure 4G–I). At the same time, it was shown that the CCRC-M35 epitope was localised mainly in the cell walls of the endodermis (Figure 4A) and was found as dots in the cell walls and infection thread walls of *M. truncatula* nodules (Figure 4B), but was absent in *P. sativum* nodules (Figure 4C).

The epitope of the backbone of RG-I, detected by the CCRC-M36 antibody, in the wild-type nodules of *M. truncatula* was found in some cell walls in the meristem (Figure 4D), was well identified in the cell walls and infection thread walls in the infection zone (Figure 4E) and mainly disappeared from cell walls, remaining in the infection thread walls in the nitrogen fixation zone (Figure 4F). In the wild-type *P. sativum* nodules, this epitope was localised in the cell walls and infection thread walls in the meristem (Figure 4G) and the infection zone (Figure 4H), while in the nitrogen fixation zone, it remained mainly in the infection thread walls (Figure 4I). Immunogold study showed the presence of CCRC-M35 (Figure S2A,C) and CCRC-M36 (Figure S2B,D) labels in small amounts in the cell walls and infection thread walls in wild-type *M. truncatula* nodules.

In *P. sativum* mutants characterised by a block in the early stages of infection development, the CCRC-M36 epitope was found in the cell walls and infection thread walls, but the label was less abundant compared to the wild type (Figure 4J,K). In the early senescent mutant SGEFix^–^-3 (*sym26*), this epitope was completely absent in degrading infected cells (Figure 4L).

### 2.5. Rhamnogalacturonan I (Linear (1-5)-α-L-Arabinan)

**Figure 5:**
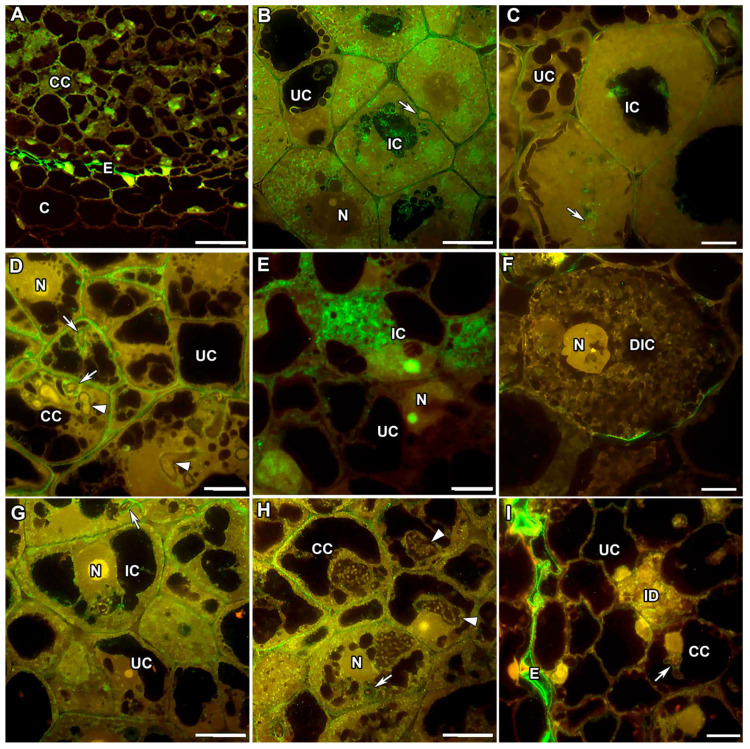
Fluorescent immunolocalisation of the (1→5)-α-L-arabinan side chain of rhamnogalacturonan I labelled with LM6-M in nodules from wild-type and mutant *Medicago truncatula* (**G**–**I**) and *Pisum sativum* (**A**–**F**). The secondary antibody used was the goat anti-rat IgG monoclonal antibody conjugated with Alexa Fluor 488. (**A**) Endodermis of the wild-type SGE; (**B**) infection zone of the wild-type SGE; (**C**) nitrogen fixation zone of the wild-type SGE; (**D**) colonised cells of the mutant SGEFix^−^-2 (*sym33-3*); (**E**) infected cell of the mutant Sprint2Fix^−^ (*sym31*); (**F**) senescence zone of the mutant SGEFix^−^-3 (*sym26*); (**G**) infection zone of the wild-type A17; (**H**) infection zone of the mutant *efd–1*; (**I**) colonised cells of the mutant TR3 (*ipd3*). C—cortex, E—endodermis, CC—colonised cell, IC—infected cell, UC—uninfected cell, DIC—degraded infected cell, N—nucleus, ID—infection droplet; arrows indicate infection threads, triangles indicate infection thread without signal. Bars = 10 μm.

**Figure 6:**
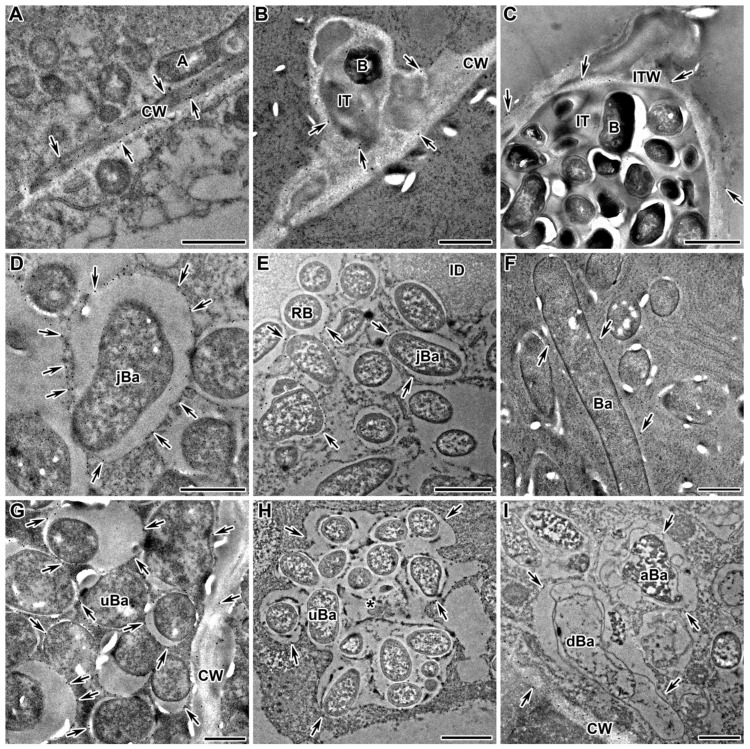
Immunogold localisation of the (1→5)-α-L-arabinan side chain of rhamnogalacturonan I labelled with LM6-M in nodules from wild-type and mutant *Medicago truncatula* (**B**,**C**,**F**,**G**) and *Pisum sativum* (**A**,**D**,**E**,**H**,**I**). The secondary antibody used was the goat anti-rat IgG monoclonal antibody conjugated to 10 nm diameter colloidal gold. (**A**) Cell wall in the wild-type SGE; (**B**) infection thread in the wild-type A17; (**C**) infection thread in the mutant TR3 (*ipd3*); (**D**) juvenile symbiosomes in the wild-type SGE; (**E**) juvenile symbiosomes in the mutant SGEFix^−^-1 (*sym40-1*); (**F**) mature symbiosomes in the wild-type A17; (**G**) undifferentiated symbiosomes in the mutant *dnf1–1*; (**H**) multibacteroid symbiosomes in the mutant SGEFix^−^-1 (*sym40-1*); (**I**) symbiosomes with abnormal and degraded bacteroids in the mutant SGEFix^−^-1 (*sym40-1*). CW—cell wall, A—amyloplast, IT—infection thread, ITW—infection thread wall, B—bacterium, Ba—mature bacteroid, jBa—juvenile bacteroid, uBa—undifferentiated bacteroid, aBa—abnormal bacteroid, dBa—degraded bacteroid, *—multibacteroid symbiosomes; arrows indicate gold particles. Bars (**A**–**C**,**E**,**H**) = 1 μm, (**D**,**F**,**G**,**I**) = 500 nm.

In the wild-type nodules of both *M. truncatula* and *P. sativum*, the presence of the epitope of the linear arabinan side chain of RG-I, recognised with the monoclonal antibody LM6-M, was localised in the cell walls of infected, uninfected, and colonised cells and in the infection thread walls (Figure 5), as well as in the cell walls of degraded infected cells (Figure 5F). Immunogold analysis confirmed the distribution of the arabinan epitope in the cell walls and infection thread walls (Figure 6A,B). Arabinan RG-I was found in the infection thread walls in the nodules of the mutants in the orthologous genes *IPD3* and *Sym33* (Figure 5D,I and Figure 6C). In addition, the LM6-M label was observed in the cell walls of the endodermis in postmeristematic areas (Figure 5A,I).

The LM6-M antibody used in the assay is known to cross-react with the arabinogalactan protein (AGP) epitope. At the same time, the dependence of the distribution of arabinan, determined using this antibody, on the stage of nodule development was shown (Figure 5B,C). In addition to the localisation of the LM6-M label in the cell walls, this antibody marked the symbiosomes with juvenile bacteroids in the infected cells in the nodules of the *P. sativum* wild-type SGE (infection and early nitrogen fixation zone) (Figure 5B and Figure 6D), the mutant SGEFix^–^-1 (*sym40-1*) (Figure 6E), and in the nodules of the *M. truncatula* wild-type A17 (infection and early nitrogen fixation zone) (Figure 5G) and the mutant *efd–1* (Figure 5H). In the *P. sativum* mutant SGEFix^–^-1 (*sym40-1*), multibacteroid symbiosomes, containing undifferentiated bacteroids, are often formed; they were also labelled with the LM6-M antibody (Figure 6H). In the mutants of *P. sativum* Sprint-2Fix^−^ (*sym31*) and *M. truncatula dnf1–1,* characterised by undifferentiated bacteroids, the LM6-M label was also present in symbiosomes (Figure 5E and Figure 6G, respectively). Later, in the nitrogen fixation zone, the label in symbiosomes remained visible only at the ultrastructural level, which was shown for the nodules of the wild-type *P. sativum* SGE (Figure 5C) and *M. truncatula* A17 (Figure 6F). In addition, it was shown that the arabinan epitope label disappeared in the *P. sativum* mutant SGEFix^–^-3 (*sym26*), which is characterised by early senescence (Figure 5F), and immunogold analysis demonstrated a low amount of the LM6-M label in the abnormally differentiated bacteroids in the *P. sativum* mutant SGEFix^–^-1 (*sym40-1*) (Figure 6I). In the mutants of *P. sativum* SGEFix^−^-2 (*sym33-3*) and *M. truncatula* TR3 (*ipd3*), characterised by the absence of bacterial release from infection threads, the RG-I epitope labelled with the LM6-M antibody was localised exclusively in the cell walls (Figure 5D,I, respectively).

### 2.6. Xylogalacturonan

**Figure 7:**
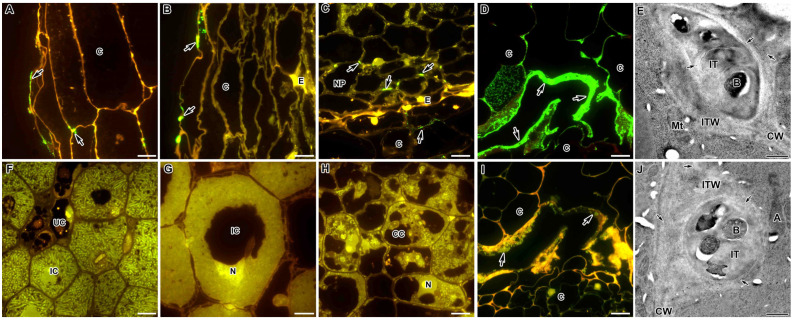
Fluorescent immunolocalisation of xylogalacturonan labelled with LM8 (**A**–**D**) and feruloylated-(1→4)-β-D-arabinan labelled with LM12 (**F**–**I**) in nodules from wild-type and mutant *Medicago truncatula* (**A**,**C**–**F**,**H**–**J**) and wild-type *Pisum sativum* (**B**,**G**). The secondary antibody used was the goat anti-rat IgG monoclonal antibody conjugated with Alexa Fluor 488. Immunogold localisation of xylogalacturonan labelled with LM8 (**E**) and feruloylated-(1→4)-β-D-arabinan labelled with LM12 (**J**) in nodules from the *M. truncatula* wild-type A17. The secondary antibody used was the goat anti-rat IgG monoclonal antibody conjugated to 10 nm diameter colloidal gold. (**A**) Cortex of the wild-type A17; (**B**) cortex of the wild-type SGE; (**C**) cortex and nodule parenchyma of the mutant *efd–1*; (**D**) cortex of the mutant TR3 (*ipd3*); (**E**) infection thread in the wild-type A17; (**F**) nitrogen fixation zone of the wild-type A17; (**G**) nitrogen fixation zone of the wild-type SGE; (**H**) infection zone of the mutant *efd–1*; (**I**) cortex of the mutant TR3 (*ipd3*); (**J**) infection thread in the wild-type A17. C—cortex, E—endodermis, NP—nodule parenchyma, CC—colonised cell, IC—infected cell, N—nucleus, CW—cell wall, Mt—mitochondrion, A—amyloplast, IT—infection thread, ITW—infection thread wall, B—bacterium; arrows indicate signal, small arrows indicate gold particles. Bar (**D**) = 50 µm, (**A**–**C**,**F**–**I**) = 10 μm, (**E**,**J**) = 500 nm.

An immunocytochemical study of the localisation of xylogalacturonan using the monoclonal antibody LM8 in the nodules of *M. truncatula* and *P. sativum* demonstrated the absence of this pectin in the cell walls and infection thread walls. It was observed only in the outer epidermal cells, where this polysaccharide was located either in a thin layer or dots in the outer cell wall (Figure 7A,B, respectively). In the *M. truncatula* mutant *efd-1*, the LM8 label appeared at the three-cell junctions of the inner nodule parenchyma (Figure 7C), while in the mutant TR3 (*ipd3*), the accumulation of the LM8 label occurred in a thick layer in the cortex (Figure 7D). However, immunogold analysis showed that the LM8 label was still present in the infection thread walls in a small amount (Figure 7E).

### 2.7. Feruloylated-(1→4)-β-D-Arabinan

The feruloylated polysaccharide recognised with the LM12 antibody was completely absent in the wild-type nodules of *M. truncatula* and *P. sativum* (Figure 7F,G). This epitope was also absent in *M. truncatula* and *P. sativum* mutants (Figure 7H). However, the *M. truncatula* mutant TR3 (*ipd3*) accumulated the feruloylated polysaccharide (Figure 7I). Immunogold analysis showed a very rare LM12 label in the cell walls and infection thread walls in the wild-type nodules (Figure 7J).

### 2.8. Fucosylated Xyloglucan

**Figure 8:**
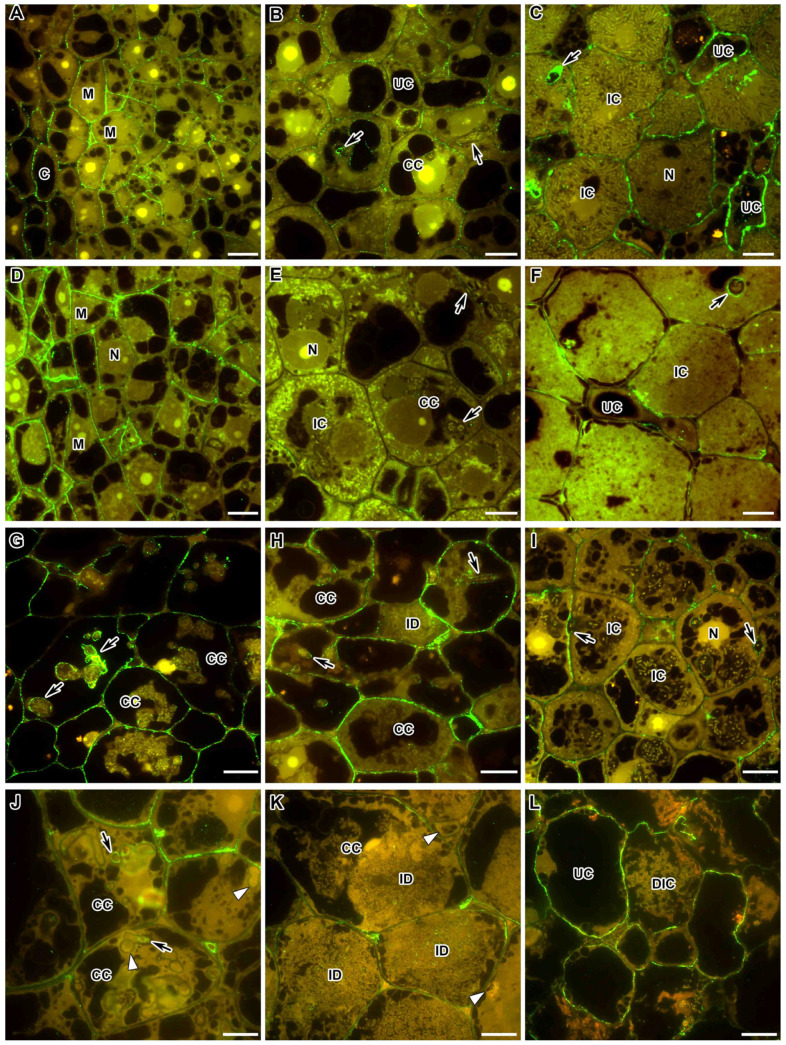
Fluorescent immunolocalisation of fucosylated xyloglucan labelled with CCRC-M1 in nodules from wild-type and mutant *Medicago truncatula* (**A**–**C**,**G**–**I**) and *Pisum sativum* (**D**–**F**,**J**–**L**). The secondary antibody used was the goat anti-mouse IgG monoclonal antibody conjugated with Alexa Fluor 488. (**A**) Meristem of the wild-type A17; (**B**) infection zone of the wild-type A17; (**C**) nitrogen fixation zone of the wild-type A17; (**D**) meristem of the wild-type SGE; (**E**) infection zone of the wild-type SGE; (**F**) nitrogen fixation zone of the wild-type SGE; (**G**) colonised cells of the mutant TR3 (*ipd3*); (**H**) infection zone on the mutant *efd–1*; (**I**) infection zone in the mutant *dnf1–1*; (**J**) colonised cells of the mutant SGEFix^−^-2 (*sym33-3*); (**K**) infection zone of the mutant SGEFix^−^-1 (*sym40-1*); (**L**) senescence zone of the mutant SGEFix^−^-3 (*sym26*). C—cortex, M—meristematic cell, CC—colonised cell, IC—infected cell, UC—uninfected cell, DIC—degraded infected cell, N—nucleus, ID—infection droplet; arrows indicate infection threads, triangles indicate infection thread without signal. Bar = 10 μm.

To study the localisation and distribution of fucosylated xyloglucan (XG), the monoclonal antibody CCRC-M1 was used, which recognises α-Fuc-(1,2)-β-Gal glycan epitope, an epitope commonly found in the side chains of XG [36]. In the wild-type nodules of both species, in the meristem, the epitope of fucosylated XG was localised in the cell walls (Figure 8A,D). In the infection zone in the *M. truncatula* nodules, the CCRC-M1 label became less intense and was located as dots in the cell walls (Figure 8B), while in the nitrogen fixation zone, it accumulated in the cell walls, especially in uninfected cells and in the infection thread walls (Figure 8C). In the infection zone of *P. sativum* nodules, the CCRC-M1 label was almost absent (Figure 8E). In the nitrogen fixation zone, the CCRC-M1 epitope was found in small amounts in some cell walls, but it was present in the infection thread walls (Figure 8F).

In the *M. truncatula* mutants with blocks at an early stage of development, the localisation of the CCRC-M1 epitope almost coincided with that in the wild type (Figure 8G–I); however, in colonised cells, the CCRC-M1 label was more abundant (Figure 8G,H). In the mutant *dnf1–1* with undifferentiated bacteroids, the CCRC-M1 label was significantly reduced in the cell walls (Figure 8I).

In the *P. sativum* mutant SGEFix^–^-2 (*sym33-3*) with the absence of bacterial release from the infection threads, the epitope of fucosylated XG was found in the thickened cell walls and in the walls of some infection threads (Figure 8J). In the mutant SGEFix^–^-1 (*sym40-1*), which is characterised by hypertrophied infection droplets, the fucosylated XG label was found in the cell walls of colonised and infected cells, but was absent in the infection thread walls (Figure 8K). In the early senescent mutant SGEFix^–^-3 (*sym26*), the CCRC-M1 label was abundant in the cell walls of degraded and uninfected cells (Figure 8L).

### 2.9. Arabinogalactan Protein

**Figure 9:**
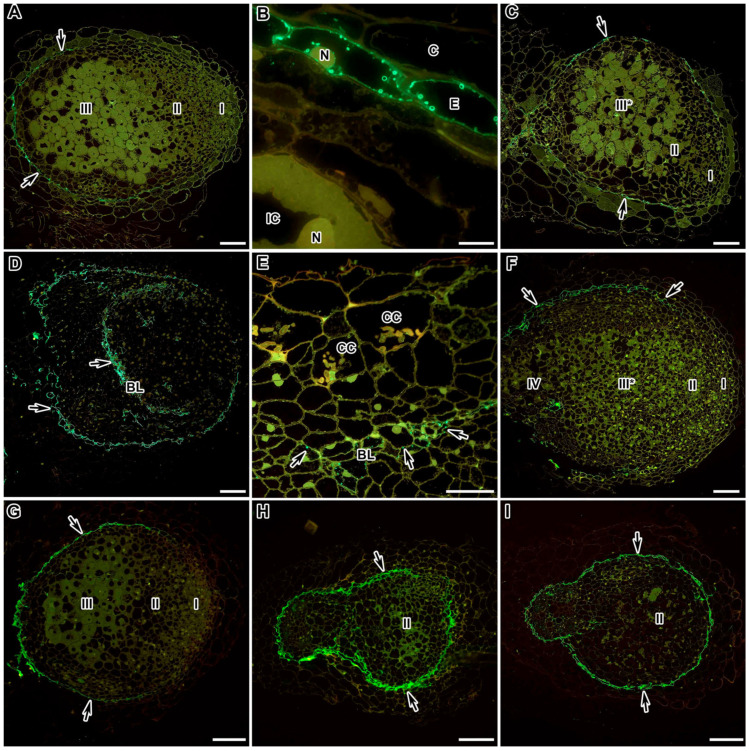
Fluorescent immunolocalisation of arabinogalactan protein labelled with JIM13 in nodules from wild-type and mutant *Medicago truncatula* (**G**–**I**) and *Pisum sativum* (**A**–**F**). The secondary antibody used was the goat anti-rat IgG monoclonal antibody conjugated with Alexa Fluor 488. (**A**) Section of a nodule of the wild-type SGE; (**B**) endodermis of the wild-type SGE; (**C**) section of a nodule of the mutant SGEFix^−^-1 (*sym40-1*); (**D**) section of a nodule of the mutant SGEFix^−^-2 (*sym33-3*); (**E**) “barrier layer” in the nodule of the mutant SGEFix^−^-2 (*sym33-3*); (**F**) section of a nodule of the mutant SGEFix^−^-3 (*sym26*); (**G**) section of a nodule of the wild-type A17; (**H**) section of a nodule of the mutant *efd–1*; (**I**) section of a nodule of the mutant TR3 *(ipd3)*. I—meristem, II—infection zone, III—nitrogen-fixation zone, III*—zone corresponding to the nitrogen fixation zone of the wild type, IV—senescence zone, C—cortex, E—endodermis, CC—colonised cell, IC—infected cell, N—nucleus, BL—“barrier layer”; arrows indicate the signal. Bars (**A**,**C**,**D**,**F**,**G**) = 100 µm, (**H**,**I**) = 50 µm, (**E**) = 20 µm, (**B**) = 5 μm.

The immunocytochemical analysis of the AGP epitope found that in the tissues of wild-type and mutant nodules of both *P. sativum* and *M. truncatula*, the JIM13-recognisable epitope was localised in the endodermal cells that constitute the “oxygen barrier” in the nodule (Figure 9). It was localised in the cell walls of the endodermis in the form of droplets on the inner surface of the cell walls (Figure 9B). In the nodules of the wild-type and mutant SGEFix^–^-1 (*sym40-1*) and SGEFix^–^-3 (*sym26*) of *P. sativum* (Figure 9A,C,F, respectively) and the *M. truncatula* wild-type A17 (Figure 9G), the JIM13-labelled AGP epitope was present in the endodermis surrounding the nodule, which was absent in the meristem (Figure 9A,C,F,G).

However, in the *M. truncatula* mutants *efd–1* and TR3 (*ipd3*) and the *P. sativum* mutant SGEFix^−^-2 (*sym33-3*), the endodermis completely surrounded the nodules, preventing them from growing. In addition, in nodules of the *P. sativum* mutant SGEFix^−^-2 (*sym33-3*), the so-called “barrier layer”, that does not allow for the infection threads to penetrate deep into the developing nodule, was observed (Figure 9D). In this “barrier layer”, an AGP epitope, recognised by the JIM13 antibody, was also observed (Figure 9E).

### 2.10. Extensin

**Figure 10:**
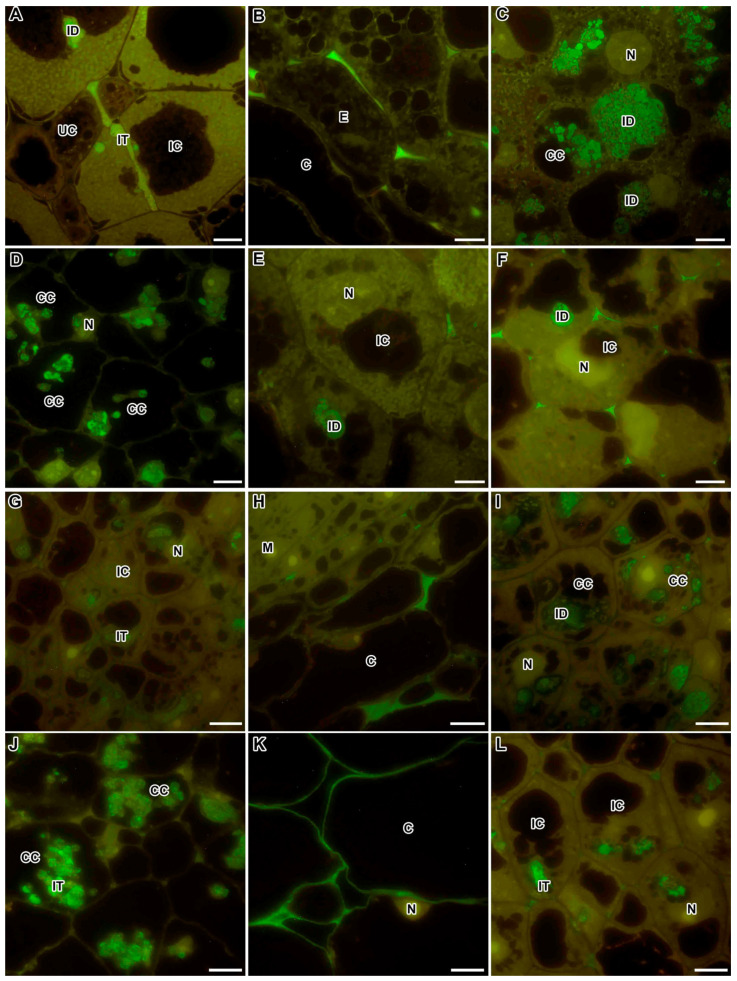
Fluorescent immunolocalisation of extensin labelled with JIM11 in nodules from wild-type and mutant *Medicago truncatula* (**G**–**L**) and *Pisum sativum* (**A**–**F**). The secondary antibody used was the goat anti-rat IgG monoclonal antibody conjugated with Alexa Fluor 488. (**A**) Nitrogen fixation zone of the wild-type SGE; (**B**) endodermis of the wild-type SGE; (**C**) infection zone of the mutant SGEFix^−^-1 (*sym40-1*); (**D**) colonised cells of the mutant SGEFix^−^-2 (*sym33-3*); (**E**) infection zone in the mutant SGEFix^−^-3 (*sym26*); (**F**) infection zone of the mutant Sprint2Fix^−^ (*sym31*); (**G**) infection zone of the wild-type A17; (**H**) endodermis of the wild-type A17; (**I**) infection zone of the mutant *efd–1*; (**J**) colonised cells of the mutant TR3 (*ipd3*); (**K**) cortex of the mutant TR3 (*ipd3*); (**L**) infection zone in the mutant *dnf1–1*. C—cortex, E—endodermis, CC—colonised cell, IC—infected cell, N—nucleus, ID—infection droplet, IT—infection thread. Bar = 10 μm.

The extensin epitope recognised by the JIM11 antibody in the wild-type nodules of *P. sativum* SGE and *M. truncatula,* A17 was deposited in the intercellular spaces of the nodule parenchyma near the endodermis (Figure 10B,H) and in the matrix of infection threads and infection droplets (Figure 10A,G). Electron microscopic analysis, conducted using the immunogold reaction, confirmed the presence of a JIM11-recognisable epitope in *P. sativum* and *M. truncatula* wild-type and mutant nodules in the matrix of infection threads (Figure S3A,D, respectively), infection droplets (Figure S3C) and intercellular space (Figure S3B). In *P. sativum* and *M. truncatula* mutants in the orthologous genes SGEFix^–^-1 (*sym40-1*) and *efd–1* (Figure 10C,I, respectively) and SGEFix^–^-2 (*sym33-3*) and TR3 (*ipd3*) (Figure 10D,J, respectively), as well as in the *P. sativum* mutant SGEFix^−^-3 (*sym26*) (Figure 10E), the extensin epitope was localised in the matrix of infection threads and droplets, as in the wild type. However, in the *M. truncatula* mutant TR3 (*ipd3*), the JIM11 label also appeared in the cell walls of the cortex (Figure 10K). In *P. sativum* and *M. truncatula* mutants with undifferentiated bacteroids Sprint-2Fix^−^ (*sym31*) and *dnf1–1* (respectively), the JIM11 label in the intercellular space was more abundant than in the wild-type nodules (Figure 10F,L, respectively).

## 3. Discussion

Plant cell walls are a specialised extracellular matrix that is primarily composed of carbohydrate polymers, serving as a dynamic support structure in plants [37]. The so-called primary cell wall, which surrounds the actively growing plant cells, is composed of cellulose, hemicelluloses, pectins, and structural glycoproteins, and contains a wide range of enzymes and other proteins that can modify the structure of cell walls or respond to internal and external stimuli [38,39,40]. Cellulose–hemicellulose networks act as the main load-bearing components of plant cell walls [41], and pectins perform important functions in wall architecture, cell growth, and tissue morphogenesis [39,42,43].

Pectin is probably one of the most complex macromolecules in nature [39,44,45,46], and it is a family of galacturonic acid-rich polysaccharides, including homogalacturonan (HG), rhamnogalacturonan-I (RG-I) and substituted galacturonan, rhamnogalacturonan-II (RG-II); some plant cell walls also contain additional substituted galacturonans known as apigalacturonan and xylogalacturonan [45,46,47].

In the formation and functioning of the symbiotic interface in the nodules of legumes, HG has been the most studied [48], which is explained by the presence of highly specific monoclonal antibodies in this polysaccharide. The HG functions in nodules, as in other plant organs, are determined by the degree of its methylation [49,50,51]. HG methylesterification is highly regulated by the plant, and pectin polysaccharides are deposited in a tissue-specific and spatiotemporal manner [39,44,52]. Studies using antibodies against highly methylesterified HG (JIM7 and LM20) or lower methylesterified HG (JIM5 and LM19) have shown that the distribution of HG with different levels of methyl ester can vary on a very small spatial scale within tissues, indicating an important role in cell development [12,51,53].

During cell differentiation, HG is synthesised in the Golgi apparatus of plants and secreted in a highly methylesterified form [39,47]. In Arabidopsis, the walls of the dividing meristematic cells and the cells of the elongation zone contain a large amount of highly methylesterified pectin, while the central cells of the resting root centre contain pectins with a low degree of methylesterification [54]. This indicates that non-growing cell walls are usually associated with de-esterified pectin, and therefore with a gel-like form, while cell growth is provided by a high degree of pectin methylesterification [42]. Highly methylesterified HG is present at all stages of nodule development [48]. During nodule development in both *M. truncatula* and *P. sativum*, uniform labelling with the JIM7 antibody, which recognises highly methylesterified HG, has previously been demonstrated in both the cell walls and the infection thread walls [12,55].

In the present study, HG appeared in the cell walls and infection thread walls in both the infection (Figure 1A,B) and nitrogen fixation (Figure 1C,D) zones in *M. truncatula* and *P. sativum* nodules (Table 1). Using the early senescent mutant SGEFix^–^-3 (*sym26*), it was demonstrated that in the senescence zone, methylesterified HG disappeared from the cell walls of degrading infected cells, while remaining in the cell walls of uninfected cells (Figure 1L). Since the JIM7 and LM20 antibodies differ in the number of methyl residues recognised on the backbone of the HG macromolecule, it can be postulated with confidence that for both studied species, a uniform distribution of highly methylesterified HG was confirmed in the cell walls, corresponding to isodiametrically growing cells, and in the infection thread walls [12]. At the same time, some deviations in the degree of HG methylation were observed in mutants exhibiting blocks in the development of infection structures, in particular infection threads. Thus, in the mutants for the orthologous genes *EFD* and *Sym40* in the meristem, highly methylesterified HG was either completely absent or was determined at the three-cell junctions (Figure 1G,H, respectively). In the mutants for the orthologous genes *IPD3* and *Sym33*, highly methylesterified HG was not detected in the cell walls and the infection thread walls (Figure 1E,F, respectively). The last observation can be explained by the abnormal thickening of the infection thread walls and cell walls, which is characteristic of these mutants (Table 1) [12,34,35,56].

Once in the cell wall, methylesterified pectins can undergo demethylesterification under the action of pectin methylesterases and turn into unesterified pectins [43,50,52,57]. Unesterified pectins can either bind Ca^2+^ to form the so-called “egg box” dimeric structures that contribute to cell wall hardness [58,59,60], or they can be cleaved by pectate lyases and polygalacturonases, causing cell wall loosening [54,60,61,62,63]. It was previously shown that HG with a low degree of methylation was involved in the increase in the hardness of the cell walls and infection thread walls, especially in the case of inefficient interaction with rhizobia and the influence of abiotic stresses [34,64].

In the present study, demethylesterified HG, recognised by the LM19 antibody, and HG, bound into a dimeric “egg box” structure with Ca^2+^, recognised by the 2F4 antibody, demonstrated a similar pattern of localisation (Table 1). They appeared in the meristem and infection zone in the nodules of both species at the three-cell junctions (Figure 2A,B and Figure 3A,B,D,E). Only in the nitrogen fixation zone, it was distributed throughout the entire thickness of the cell wall of infected cells (Figure 2C,D and Figure 3C). Surprisingly, in the nodules of the *P. sativum* mutant SGEFix^–^-2 (*sym33-3*), the intensity of the LM19 fluorescent label was high in the walls of colonised cells. It is possible that this distribution pattern for low methylesterified HG may be caused by the activation of cellular defence responses in this mutant [34,35].

In the histological zones of the nodules of both species, demethylesterified HG and HG bound by Ca^2+^ were found in the infection thread walls, especially in places where the infection threads crossed the cell walls (Figure 2 and Figure 3; Table 1). Recently, the coordinated action of one of the symbiosis-specific pectin methylesterases (SyPME1) and nodulation pectate lyase (NPL) at the sites of penetration of infection threads and their transcellular passages has been described. Their coordinated work contributes to spatially limited changes in the HG in the intercellular interface, which lead to the establishment of a specific apoplastic compartment, where bacteria enter the host cells. This process ensures successful intracellular progression of infection threads in nodules [51]. In the mutants of *P. sativum* SGEFix^–^-2 (*sym33-3*) and *M. truncatula* TR3 (*ipd3*) for orthologous genes, the intensity of the LM19 mark increased in the thickened infection thread walls (Figure 2E,F). Previously, quantitative analysis demonstrated for the mutant SGEFix^–^-2 (*sym33-3*) (but not for the mutant TR3 (*ipd3*)) an increase in demethylesterified HG recognised with the JIM5 antibody that coincides with the induction of defence reactions (activation of peroxidases and suberinisation of the cell walls and infection thread walls) [12,34,35]. In addition, in contrast to TR3 (*ipd3*), in SGEFix^–^-2 (*sym33-3*), there was no calcium-bound HG label (Figure 3H,I) in the walls of some infection threads.

Another common cell wall pectin is rhamnogalacturonan I (RG-I) [48]. It is an extremely branched pectin with numerous and diverse side chains: arabinan, galactan, type I and II arabinogalactan, or fucosyl chains [65,66]. The RG-I backbone plays an important role in the integrity and functioning of the cell wall, since its degradation through the expression of hydrolases leads to morphological changes [63]. Predominant localisation of the CCRC-M36 label, which binds to at least three unbranched disaccharide repeats in the nodules of both legume species, clearly indicates that long-chain repeats of the unbranched RG-I backbone are common in the nodules (Figure 4; Table 1). This epitope is characterised by the localisation in the cell walls of colonised and newly infected cells in the infection zone in the nodules of *M. truncatula* and *P. sativum* (Figure 4E,H); in the *P. sativum* nodules, the additional CCRC-M36 label was also found in the meristematic cell walls (Figure 4G). In the nitrogen fixation zone, the RG-I backbone marker decreased significantly, remaining in the infection thread walls (Figure 4I). In the senescence zone, the epitope of the RG-I backbone disappeared completely, which was confirmed in the early senescent mutant of *P. sativum* SGEFix^–^-3 (*sym26*) (Figure 4L). In the other mutants of *P. sativum*, the intensity of the CCRC-M36 label was significantly reduced (Figure 4J,K).

RG-I is the most structurally heterogeneous of the pectins, and the abundance and composition of RG-I side chains vary significantly between different cell types, developmental stages, and species [67,68]. Arabinan and galactan side chains of RG-I often have different or mutually exclusive localisation patterns; in general, arabinan tends to predominate in juvenile cells, while expanding cells tend to contain more galactan [69,70,71].

We have previously shown a certain species-specific localisation and distribution of the linear galactan side chain of RG-I in the nodules of *M. truncatula* and *P. sativum* [12]. Thus, fluorescence microscopy of the epitope of the linear (1→4)-β-D-galactan side chain of RG-I, recognised by the LM5 antibody, showed that the label was observed only in the cell walls of the meristem, endodermis, and phloem in the symbiotic nodules in *M. truncatula* and *P. sativum* wild types and mutants; however, in the nodules of all *P. sativum* genotypes, the LM5 label was also determined in the infection thread walls (Table 1) [12].

The RG-I arabinan side chain epitope recognised by the LM6-M antibody was found not only in the cell walls (Figure 6A) and infection thread walls (Figure 6B,C), but it also labelled symbiosomes in the infected cells (Figure 6D–I). Type II arabinogalactan side chains that occur in RG-I also occur on the cell surface of AGP proteoglycans, as has been shown for the arabinan epitope recognised by LM6 [72]. When analysing the localisation of the RG-I arabinan side chain epitope using the LM6-M antibody, the dependence of the arabinan distribution on the stage of nodule development was shown (Figure 5A–C; Table 1). In the nodules of both species, the LM6-M antibody labelled juvenile symbiosomes in infected cells from the infection and early nitrogen fixation zones (Figure 5B,G,H and Figure 6D,E) and undifferentiated symbiosomes in mutants (Figure 5E and Figure 6G,H). Later, in the nitrogen fixation zone, the amount of the label in symbiosomes was reduced, which was shown for the wild-type nodules (Figure 5C and Figure 6F) and mutants SGEFix^–^-3 (*sym26*), characterised by early senescence (Figure 5F) and SGEFix^–^-1 (*sym40-1*), forming abnormal bacteroids (Figure 6I). In the case when there was no bacterial release in the nodules of mutants of both species, the LM6-M label was localised only in the cell walls and the infection thread walls (Figure 5D,I and Figure 6C).

AGP is one of the most complex families of macromolecules found in plants. They are involved in many processes involved in plant growth and development [73,74], but their exact mode of action is unknown [11,23,75]. In this work, the tissue and subcellular localisation of AGP in effective and ineffective nodules of *P. sativum* and *M. truncatula* revealed certain differences in the localisation of arabinan epitopes (cross-linked with the arabinan side chain of RG-I), which indicates the specific role of individual AGPs in the development of symbiosomes.

Among the extensive family of pectins, two other substituted galacturonans, xylogalacturonan and apigalacturonan, have a more limited distribution [47]. Xylogalacturonans, which are based on galacturonic acid and xylose residues, are most abundant in reproductive tissues, although xylogalacturonan has also been found in the stems and leaves of Arabidopsis. Xylogalacturonan has not been previously studied in symbiotic nodules. It has previously been demonstrated that the LM8 epitope is the first cell wall epitope identified that is specifically associated with a plant cell division process that leads to complete cell separation [76]. Probably, its localisation in the outer cell walls is also associated with the process of cell detachment from the surface (Figure 7A,B), and this process is aggravated in mutants (Figure 7C,D).

There is another type of linkage among cell wall polysaccharides, which is ester linkages, formed with the help of the phenolic compound ferulic acid, between the median lamella pectin and the cell wall xyloglucan. Studies on Chinese water chestnut, asparagus, and sugar beet have shown that ferulic acid residues that cross-link RG-I arabinose and xylan backbone of arabinoxylan (hemicellulose) may play an important role in cell-to-cell adhesion [77,78,79,80]. The role of such a relationship between polysaccharides and the presence of ferulated polysaccharides in nodules has not been studied. However, phenolic compounds play a certain role in defence reactions in *P. sativum* nodules [34,35]. Electron microscopic examination showed the presence of trace amounts of ferulated polysaccharide in cell walls in the nodules (Figure 7J), which may indicate the involvement of ferulic acid in ester bonds between the cell wall polysaccharides [80]. The cell wall cross-linking process can provide cells with mechanical strength to withstand the pressure that is placed on cell walls during plant growth, including infection thread growth, and this is likely required for normal cell wall expansion and plant growth [80,81].

Xyloglucan (XG) is thought to crosslink cellulose microfibrils, while pectins such as HG and RG form a structurally diverse glue that provides flexibility or hardness depending on chemical modifications [38]. Fucosylation of XG is of great importance. Thus, when characterising the first mutant with an altered cell wall in Arabidopsis *mur1*, which is characterised by dwarfism and reduced stem strength, up to 98% reduction in the content of fucose in cell walls was found [82,83]. In the present study, it was shown that in the nodules of both species, fucosylated XG was characteristic of the cell walls of meristematic cells (Figure 8A,D), infected cells (Figure 8B,C,F), and uninfected cells in the nodules of *M. truncatula* (Figure 8C; Table 1). At the same time, the intensity of the label in *M. truncatula* mutants blocked at the early stages of development was higher (Figure 8G,H). This epitope was also present in the infection thread walls (Figure 8B,C,F-J), but only in the *P. sativum* mutant SGEFix^–^-2 (*sym33-3*), which is characterised by suberinisation of the cell walls and infection thread walls [34]; the CCRC-M1 mark was not observed in the walls of some infection threads (Figure 8J).

In addition to carbohydrate polymers, plant cell walls also contain structural proteins, such as hydroxyproline-rich glycoprotein, proline-rich and glycine-rich protein, and arabinogalactan protein (AGP), which change the physical and chemical properties of the cell wall in response to various ontogenetic signals and environmental influences [73,84,85].

The AGP epitope recognised by the JIM13 antibody is involved in the differentiation of the elements of the vascular system [86,87], and it exhibits a temporal expression pattern. In addition, the JIM13-recognised epitope is localised on the surface of the cells that form the epidermis [88] and in the cells of the abscission zone during fruit abscission [89]. There is an association of the JIM13 epitope with the organisation of cortical microtubules [90], the formation of the cell wall of labyrinthine invaginations of transfer cells in symbiotic nodules of *P. sativum* [91], and participation as a signalling molecule in the regulation of the production of some compounds [92]. Thus, AGP, in particular, the JIM13-recognised epitope, has a spatiotemporal pattern and can be used as a specific cell surface marker.

An immunogold study of *P. sativum* nodules has shown that the walls and plasma membranes of nodule endodermal cells carried an abundant JIM13 label [93], which was confirmed with immunofluorescent analysis (Table 1). In addition, the JIM13 label was located in the form of two arcs with a continuous layer of cells in the root, but did not affect the meristem at the top of the nodule. The observed localisation pattern of the JIM13 epitope coincides with the localisation of suberin in wild-type pea nodules [34]. As is known, the walls of mature endodermal cells are suberinised during development with the formation of a physiological barrier to the entry of water and soluble compounds. In younger cells, in which the JIM13 antigen was not yet detected at the light microscopy level; the appearance and accumulation of the label in vesicles near the plasma membrane were shown via electron microscopy [93]. Therefore, the AGP epitope recognised by JIM13 is a useful molecular marker for the differentiation of endodermal cells, in which wall modifications are not yet visible. In addition, the endodermis serves as a defence against the penetration of pathogenic microbes. Therefore, both suberinisation [34] and accumulation of the JIM13 antigen in endodermal cells completely surrounding the nodules of the *M. truncatula* mutants *efd–1* and TR3 (*ipd3*) and *P. sativum* mutant SGEFix^–^-2 (*sym33-3*) and the formation of a “barrier layer” in the *P. sativum* mutant SGEFix^–^-2 (*sym33-3*) with hypertrophied “locked” infection threads can be attributed to defence reactions.

Extensins are cell wall proteins belonging to the superfamily of hydroxyproline-rich glycoproteins and are known to be involved in many processes during plant growth and development. In addition, these proteins are involved in the interactions between plants and pathogen [5] and symbiotic [11] microorganisms. During legume–rhizobial symbiosis, arabinogalactan protein-extensins are mainly accumulated in the walls of infected cells, in the walls and matrix of infection threads [13,94], and on symbiosome membranes [12,55], which indicates their important role in nodule development. In addition, the potential involvement of extensins in symbiotic interactions has been reported [91,95]. When studying the symbiotic interaction of *P. sativum* with *Rhizobium leguminosarum*, it was shown that antibodies LM1 and LM3 [96], which recognise extensin epitopes, labelled the matrix of infection threads as strongly as MAC265 [91,95]. The involvement of the JIM11 epitope was previously revealed in the mycorrhizal symbiosis of the orchid plant *Dendrobium officinale* [97].

The extensin epitope recognised by the JIM11 antibody in the *P. sativum* wild-type nodules SGE and *M. truncatula* A17 is deposited in the intercellular spaces of the nodule parenchyma near the endodermis (Figure 10B,H), in the matrix of infection threads and infection droplets (Figure 10; Table 1), in contrast to the extensin LM1 epitope, which was also deposited in the cell walls of the nodule cortex and the parenchymal cell walls [95]. The LM1 label was continuous throughout the nodule, from the mature base to the infection zone bordering the meristem. However, the label was absent in meristematic and infected cells [95]. When exposed to aluminium, the quantity of extensins recognised by the LM1 antibody was also sharply increased, especially in the infection thread matrix and in the intercellular space [95]. We have shown that during the development of ineffective symbiosis, extensins (JIM11 label) appear in the cell walls of nodule cortex cells in the *M. truncatula* mutant TR3 (*ipd3*) with no bacterial release into plant cells (Figure 10K) and are accumulated in the intercellular space in mutants of both species with undifferentiated symbiosomes Sprint2Fix^−^ (*sym31*) and *dnf1-1* (Figure 10F,L, respectively).

## 4. Materials and Methods

### 4.1. Plant Material

*Pisum sativum* L. and *Medicago truncatula* Gaertn. ineffective (Fix^−^) mutants blocked at different stages of nodule development and corresponding wild types were used (Table 2).

### 4.2. Bacterial Strains, Inoculation and Plant Growth Conditions

Seeds were sterilised with concentrated sulphuric acid for 30 min and washed with sterile water 10 times. In all experiments, *P. sativum* plants were inoculated with *Rhizobium leguminosarum* bv. *viciae* strain 3841 [111]. *M. truncatula* plants were inoculated with *Sinorhizobium meliloti* strain 490, constitutively expressing a mCherry fluorescent protein (a derivative of the pHC60 (tetR) plasmid [112], in which the GFP coding sequence was replaced with the mCherry coding sequence (J. Fournier, LIPM, Toulouse, France, unpublished results) as described previously [113]. The seeds were planted in pots containing 200 mL of vermiculite and 100 mL nutrient solution without nitrogen [114], and then each seed was inoculated with 1 mL of an aqueous suspension of bacteria (10^7^–10^8^ cells). Plants were grown in a growth chamber MLR-352H (Sanyo Electric Co., Ltd., Moriguchi, Japan) under controlled conditions: day/night, 16/8 h; temperature, 21 °C; relative humidity 75%; photosynthetic photon flux density of ~280 μmol photons m^−2^ s^−1^. For immunocytochemical analysis, three independent experiments were performed. Nodules of *P. sativum* were harvested on day 14 after inoculation (DAI). Nodules of *M. truncatula* were harvested 13 DAI for A17 and *dnf1–1*, 11 DAI for *efd–1*, and 16 DAI for TR3 (*ipd3*), depending on the nodule growth rate of each genotype. For each variant, ten nodules from different plants were analysed.

### 4.3. Sample Preparation

Nodules were harvested from roots and transferred directly into the fixative. Whole nodules were fixed in 2.5% glutaraldehyde in 0.06 M phosphate buffer at pH 7.2. Nodules were given a glancing cut on one side to allow for better penetration of the fixative. After vacuum infiltration, floating nodules were discarded, and the fixative was replaced with a fresh solution. After overnight incubation at 4 °C, nodules were dehydrated in an ascending ethanol series at −35 °C for 1 h at each step. For infiltration and polymerisation of *P. sativum* and *M. truncatula* nodules, London Resin White (Polysciences Europe, Eppelheim, Germany) and Lowicryl K4M Resin (Polysciences Europe, Eppelheim, Germany) were used, accordingly. Subsequently, specimens were gradually infiltrated with increasing concentrations of resin in the ratio 1:1, 1:2, and 1:3 mixed with ethanol (100%), and finally embedded in the corresponding resin using UV polymerisation in a Leica EM AFS2 (Leica Microsystems, Vienna, Austria) at −20 °C for 48 h in small plastic containers.

### 4.4. Fluorescence Microscopy

For fluorescence microscopy, the embedded material was cut into semi-thin sections (1 μm) on a Leica EM UC7 ultramicrotome (Leica Microsystems, Vienna, Austria). Sections were placed on glass slides SuperFrost (Menzel-Gläser, Thermo Fisher Scientific, Waltham, MA, USA). After blocking of non-specific binding sites by incubating the sections in a blocking solution (5% bovine serum albumin (BSA), 0.5% goat serum, and 0.05% cold water fish skin (CWFS)) and washing the slides with 3% BSA in PBS (2.48 g/L NaH_2_PO_4_, 21.36 g/L Na_2_HPO_4_, and 87.66 g/L NaCl; pH 7.2) during 15 min, the sections were incubated with a selected primary monoclonal antibody diluted (Table 2) In 3% BSA in PBS (pH 7.2) at 37 °C for 1 h. The samples were washed again in 3% BSA in PBS (pH 7.2) two times for 20 min each. The incubation with the secondary goat anti-rat IgG monoclonal antibody conjugated with Alexa Fluor 488 (Life Technologies, Grand Island, NY, USA) in 3% BSA in PBS (diluted 1:200) was conducted for 1 h at 37 °C. Then, samples were washed with PBS twice for 20 min each. After complete drying, sections were covered with a drop of ProLong Gold^®^ antifade reagent (Life Technologies, Grand Island, NY, USA). The sections were examined on a microscope Axio Imager.Z1 (Carl Zeiss, Oberkochen, Germany). Photos were taken using a digital video camera Axiocam 506 (Carl Zeiss, Oberkochen, Germany).

### 4.5. Transmission Electron Microscopy

The immunogold labelling was described previously [12]. Briefly, for transmission electron microscopy, gold sections, 90 nm thick, were obtained with a Leica EM UC7 ultramicrotome (Leica Microsystems, Vienna, Austria) and collected on formvar and carbon-coated nickel grids. The grids were placed in PBS for 30 min and 2.50 mM glycine for 60 min and were blocked in 1% BSA in PBS for 2 h and then washed in 0.1% acetylated BSA (BSA-C) in PBS. Sections were incubated with the primary antibody diluted (Table 2) in PBS containing 0.1% BSA-C overnight at 4 °C in a moist chamber. The sections were washed five times in PBS containing 0.1% BSA-C and Tween20 and incubated for 2 h in a moist chamber with the secondary antibody conjugated to 10 nm diameter colloidal gold (Amersham International, Little Chalfont, UK), diluted 1:50 in 0.1% BSA-C in PBS. The grids containing sections were washed four times in 0.1% BSA-C in PBS containing Tween20 and twice in water. After washing, sections were counterstained in 2% aqueous uranyl acetate for 1 h, followed by lead citrate for 1 min. Ultrathin sections of the selected area were examined using a Tecnai G2 Spirit electron microscope (FEI, Eindhoven, The Netherlands) at 80 kV. Digital micrographs were taken with a MegaView G2 CCD camera (Olympus-SIS, Münster, Germany).

### 4.6. Antibodies

Eleven primary antibodies were used for the immunodetection of cell wall antigens (Table 3).

### 4.7. Controls of the Specificity of the Immunolabelling

The specificity of the fluorescence and the immunogold labelling procedures was tested with several negative controls. Negative controls were treated either with (i) non-specific secondary antibody (goat anti-mouse or anti-rat IgG, respectively) or with (ii) gold-conjugated secondary antibody (goat anti-rat or anti-mouse IgG, respectively) without the primary antibody.

Negative controls revealed that no labelling occurred on the sections when they were treated with (i) non-specific secondary antibody (Figure S4A,C) and (ii) gold-conjugated secondary antibody without the primary antibody (Figure S4B,D). No specific label was detected, but autofluorescence was observed on both variants of control sections.

## 5. Conclusions

In this and our previous work [12], the ontogenetic changes in the symbiotic interface were revealed (Table 1). Nodules of *M. truncatula* and *P. sativum* belong to an indeterminate type with a constant meristem and pronounced zonation [4,121]. Therefore, in such nodules, one can trace the gradient of cell differentiation when they are infected with rhizobia.

The presence of galactans in the infection thread walls in the *P. sativum* nodules [12], the absence of fucosylated xyloglucan in the cell walls in the *P. sativum* nodules as well as its predominant accumulation in the cell walls of uninfected cells in the *M. truncatula* nodules, and the presence of the rhamnogalacturonan I (unbranched) backbone in the meristematic cells in *P. sativum* can be attributed to the most striking species-specific features of the symbiotic interface. The identified species-specific differences in the components of the symbiotic interface seem intriguing, given that both species belong to the same inverted repeat-lacking clade (IRLC). Further comparative analysis of the *P. sativum* and *M. truncatula* genomes should reveal the basis for these differences. It can be assumed that they are related to the formation by *P. sativum* and *M. truncatula* of symbioses with rhizobia belonging to different genera, *Rhizobium* and *Sinorhizobium* (*Ensifer*), respectively. At the same time, rhizobia in *P. sativum* and *M. truncatula* nodules differ significantly in the morphology of nitrogen-fixing bacteroids. We have previously identified differences between these two legume species in the composition of the symbiotic interface [12] as well as the organisation of the tubulin cytoskeleton [113]. The observed differences in the symbiotic interface composition further indicate the need to study each individual legume species. The results that have been obtained in numerous studies of the model species *M. truncatula* may not always be applicable to other legume species.

## Figures and Tables

**Table 1:** Modification of the plant–microbial interface in *Pisum sativum* and *Medicago truncatula* nitrogen-fixing nodules.

Nodule Zone, Tissue, Cell Type or Cell Structure		*P. sativum*	*M. truncatula*
Endodermis		Galactan RG-I [12] Arabinan RG-I AGP Extensin	Galactan RG-I [12] Arabinan RG-I AGP Extensin RG-I backbone
Meristem		HM HG Galactan RG-I [12] Arabinan RG-I XG RG-I backbone	HM HG Galactan RG-I [12] Arabinan RG-I XG
Infection zone	colonised cells	HM HG LM HG RG-I backbone Arabinan RG-I Ca^2+^-bound HG	HM HG LM HG RG-I backbone Arabinan RG-I XG
	infection thread walls	HM HG LM HG Ca^2+^-bound HG RG-I backbone Arabinan RG-I Galactan RG-I [12]	HM HG LM HG Ca^2+^-bound HG RG-I backbone Arabinan RG-I XG
	juvenile symbiosomes	Arabinan RG-I AGP with GPI [12]	Arabinan RG-I
Nitrogen fixation zone	infected cells	HM HG LM HG Ca^2+^-bound HG Arabinan RG-I RG-I backbone	HM HG LM HG Ca^2+^-bound HG Arabinan RG-I
	infection thread walls	HM HG LM HG XG RG-I backbone Arabinan RG-I Galactan RG-I [12]	HM HG LM HG XG RG-I backbone Arabinan RG-I Ca^2+^-bound HG
	mature symbiosomes	AGP with GPI [12] ↑	–
Uninfected cells		HM HG LM HG Arabinan RG-I Galactan RG-I [12]	HM HG LM HG Arabinan RG-I Galactan RG-I [12] XG
Intercellular space		Extensin	Extensin
Infection thread matrix		Extensin	Extensin

The table is based on the previous [12] and present studies. Only major compounds according to fluorescence microscopy data are given. Species-specific compounds are marked in red. HM HG—highly methylesterified homogalacturonan (labelled with the JIM7 [12] and LM20 antibodies), LM HG—low methylesterified homogalacturonan (labelled with the JIM5 [12] and LM19 antibodies). Other abbreviations are given according to the main text.

**Table 2:** Plant material used in the study.

Lines	Phenotype	References
*P. sativum*		
SGE	Wild type	[56,98]
SGEFix^−^-1 (*sym40-1*) ^a^	Hypertrophied infection droplets and infection threads, abnormal bacteroids	[56]
SGEFix^−^-2 (*sym33-3*) ^b^	Abnormal infection thread growth inside the nodule, no bacterial release ^c^	[56,99,100]
SGEFix^−^-3 (*sym26*)	Early senescence	[101,102]
Sprint-2	Wild type	[103]
Sprint-2Fix^−^ (*sym31*)	Undifferentiated bacteroids	[103,104]
*M. truncatula*		
cv Jemalong A17	Wild type	
*efd–1*	Hypertrophied infection droplets and infection threads, abnormal bacteroids	[105]
TR3 (*ipd3*)	Abnormal infection thread growth inside the nodule, no bacterial release	[106,107]
*dnf1–1*	Undifferentiated bacteroids	[108,109]

^a^ The *Sym40* gene is orthologous to the *M. truncatula EFD* gene [33]. ^b^ The *Sym33* gene is orthologous to the *M. truncatula IPD3* gene [33]. ^c^ The mutant line SGEFix^−^-2 (*sym33-3*) has a leaky phenotype, and in some cells or some nodules, bacterial release occurs [56,100,110].

**Table 3:** Primary antibodies used in the study.

Antibody	Dilution FM/IGL	Antigen/Epitope	Reference/Source
Pectins			
LM19	1:10/1:10, 1:25	Low methylesterified HG/α-GalA-(1→4)_(4)_	[68]; https://www.agrisera.com (accessed on 29 August 2023)
LM20	1:10/1:10, 1:25	High methylesterified HG/α-MeGalA-(1→4)_(4)_	[68]; https://www.agrisera.com (accessed on 29 August 2023)
2F4	1:10/1:200	Dimeric association of pectic chains through calcium ions	[115,116]; https://carbosource.uga.edu (accessed on 28 August 2023)
CCRC-M35	1:10/1:10, 1:25	Rhamnogalacturonan I (backbone)/Rha-(1→4)-GalA-(1→2)-Rha-(1→4)-GalA-(1→2)-Rha-(1→4) Requires at least two unbranched disaccharide repeats	[117]; https://www.agrisera.com (accessed on 28 August 2023)
CCRC-M36	1:10/1:10, 1:25	Rhamnogalacturonan I (backbone)/Rha-(1→4)-GalA-(1→2)-Rha-(1→4)-GalA-(1→2)-Rha-(1→4)-GalA-(1→2) Requires at least three unbranched disaccharide repeats	[117]; https://www.agrisera.com (accessed on 28 August 2023)
LM6-M	1:10/1:100	Rhamnogalacturonan-I/linear pentasaccharide in (1→5)-α-L-arabinans	[72,118]; https://www.agrisera.com (accessed on 28 August 2023)
LM8	1:10/1:10, 1:25	Xylogalacturonan/n/a	[76]; https://www.agrisera.com (accessed on 28 August 2023)
Xyloglucan			
CCRC-M1	1:10/1:10, 1:25	Fucosylated xyloglucan/α-Fuc-(1→2)-β-Gal	[117]; https://www.agrisera.com (accessed on 28 August 2023)
Other polysaccharides			
LM12	1:10/1:10, 1:25	Ferulated polysaccharides/n/a	[119]; https://www.agrisera.com (accessed on 28 August 2023)
Arabinogalactan protein			
JIM13	1:10/1:10, 1:25	Arabinogalactan protein/Β-linked glucuronic acid	[88]; https://www.agrisera.com (accessed on 28 August 2023)
Extensin			
JIM11	1:10/1:10, 1:25	Extensin glycoprotein/n/a	[120]; https://www.agrisera.com (accessed on 28 August 2023)

FM: fluorescence microscopy, IGL: immunogold labelling.

## Data Availability

Data are contained within the article as Supplementary Materials.

## Supplementary Materials

The following supporting information can be downloaded at: https://www.mdpi.com/article/10.3390/ijms241813850/s1.
