# Supplementary material for: Comparison of the Formation of Plant–Microbial Interface in Pisum sativum L. and Medicago truncatula Gaertn. Nitrogen-Fixing Nodules
Source: Int J Mol Sci. 2023 Sep 8;24(18):13850. doi: 10.3390/ijms241813850 (PMC10531038; doi:10.3390/ijms241813850)
Supplement: Supplementary file 1 [file ijms-24-13850-s001.zip › ijms-2576652-supplementary.pdf]

Electronic Supplementary Material

Comparison of the Formation of Plant–Microbial Interface in *Pisum sativum* L. and *Medicago truncatula* Gaertn. Nitrogen-Fixing Nodules

Anna V. Tsyganova [1,*], Elena V. Seliverstova [1,2] and Viktor E. Tsyganov [1]

[1] All-Russia Research Institute for Agricultural Microbiology, Laboratory of Molecular and Cell Biology, Saint Petersburg, Russia; avtsyganova@arriam.ru (A.V.T.); elena306@yandex.ru (E.V.S.); vetsyganov@arriam.ru (V.E.T.)

[2] Sechenov Institute of Evolutionary Physiology and Biochemistry of the Russian Academy of Sciences, Saint Petersburg, 194223, Russia; elena306@yandex.ru (E.V.S)

*Author for correspondence:

Anna V. Tsyganova

Tel: +7 812 4705100

E-mail: avtsyganova@arriam.ru

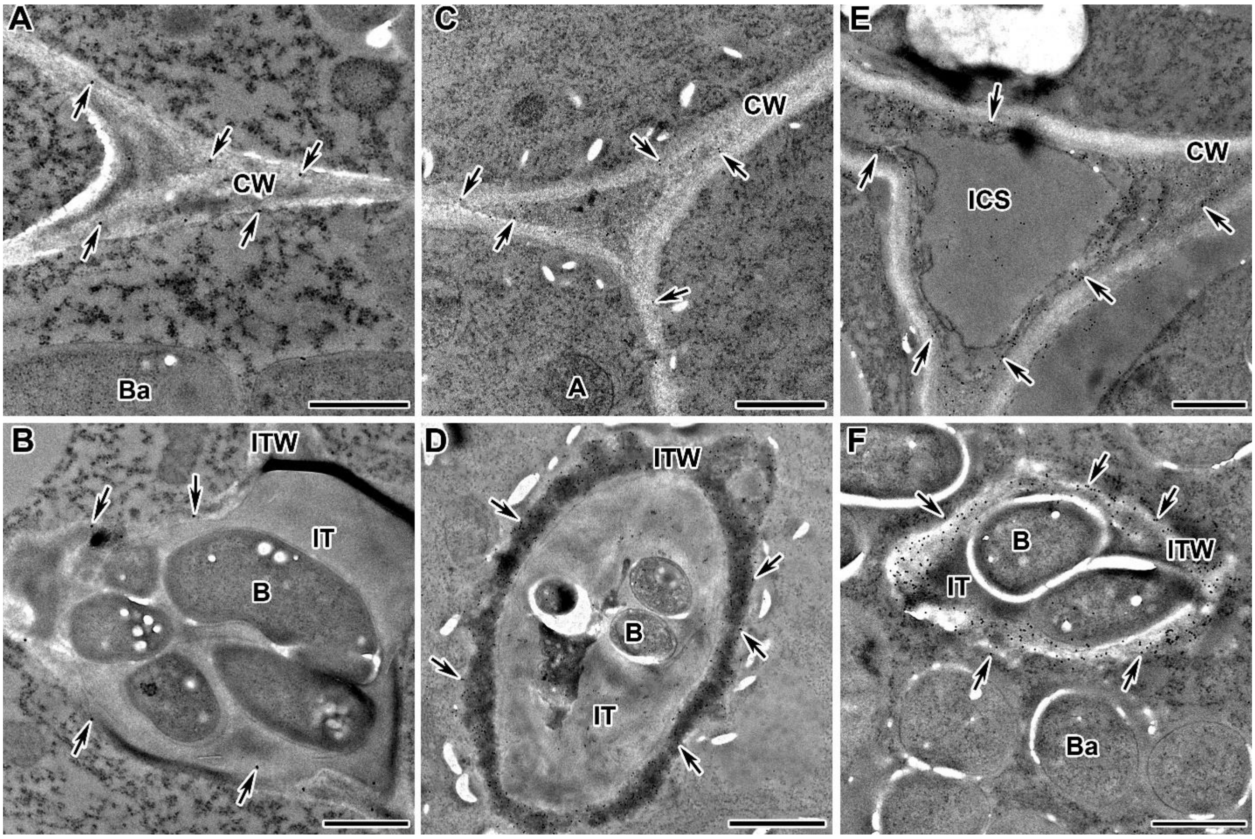

Figure S1. Immunogold localisation of highly methylesterified homogalacturonan labelled with LM20 (**A,B**), deesterified homogalacturonan labelled with LM19 (**C,D**) and the dimeric association of homogalacturonan chains through Ca^{2+} ions labelled with 2F4 (**E,F**) in nodules from wild types of *M. truncatula* (**C,D**) and *P. sativum* (**A,B,E,F**). The secondary antibody used was the goat anti-rat (**A-D**) and anti-mouse (**E,F**) IgG monoclonal antibody conjugated to 10 nm diameter colloidal gold. (**A,C,E**) Three-cell junctions; (**B,D,F**) infection threads. CW—cell wall, A—amyloplast, ICS—intercellular space, IT—infection thread, ITW—infection thread wall, B—bacterium, Ba—bacteroid; arrows indicate gold particles. Bars (**D**) = 1 μm, (**A-C,E,F**) = 500 nm.

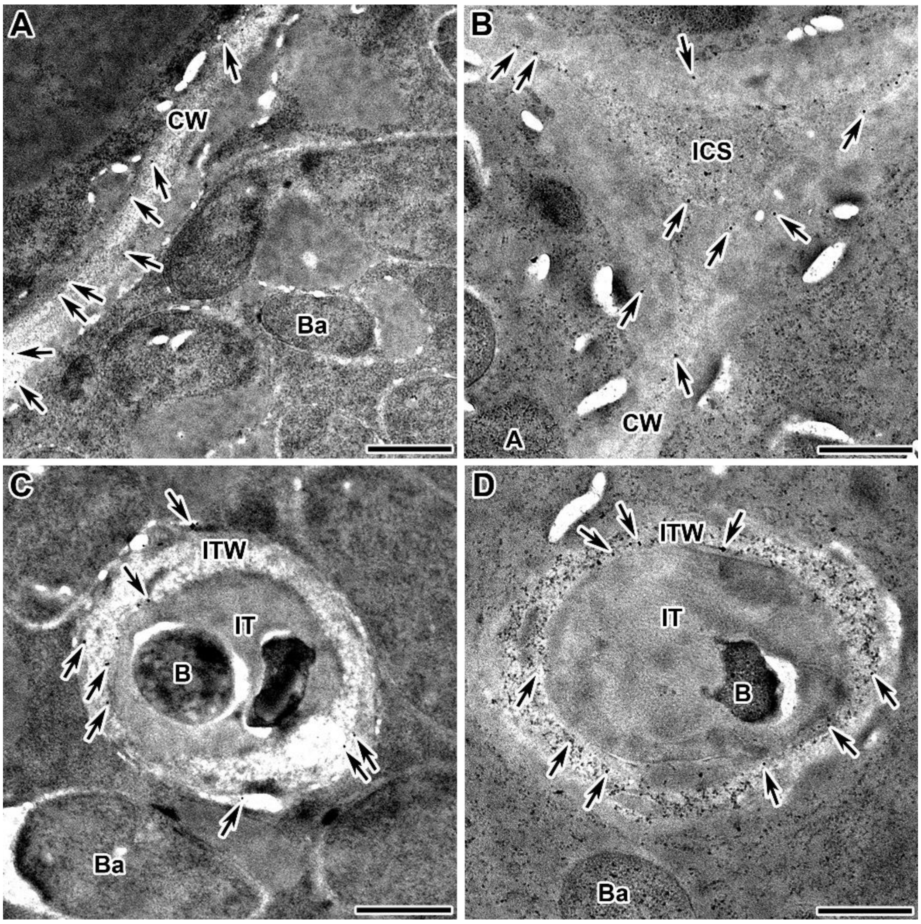

Figure S2. Immunogold localisation of the rhamnogalacturonan I backbone labelled with CCRC-M35 (A,C) and CCRC-M36 (B,D) in nodules from the wild-type *M. truncatula* A17. The secondary antibody used was the goat anti-mouse IgG monoclonal antibody conjugated to 10 nm diameter colloidal gold. (**A,B**) Cell wall and three-cell junction; (**C,D**) infection threads. CW—cell wall, A—amyloplast, ICS—intercellular space, IT—infection thread, ITW—infection thread wall, B—bacterium, Ba—bacteroid; arrows indicate gold particles. Bar = 500 nm.

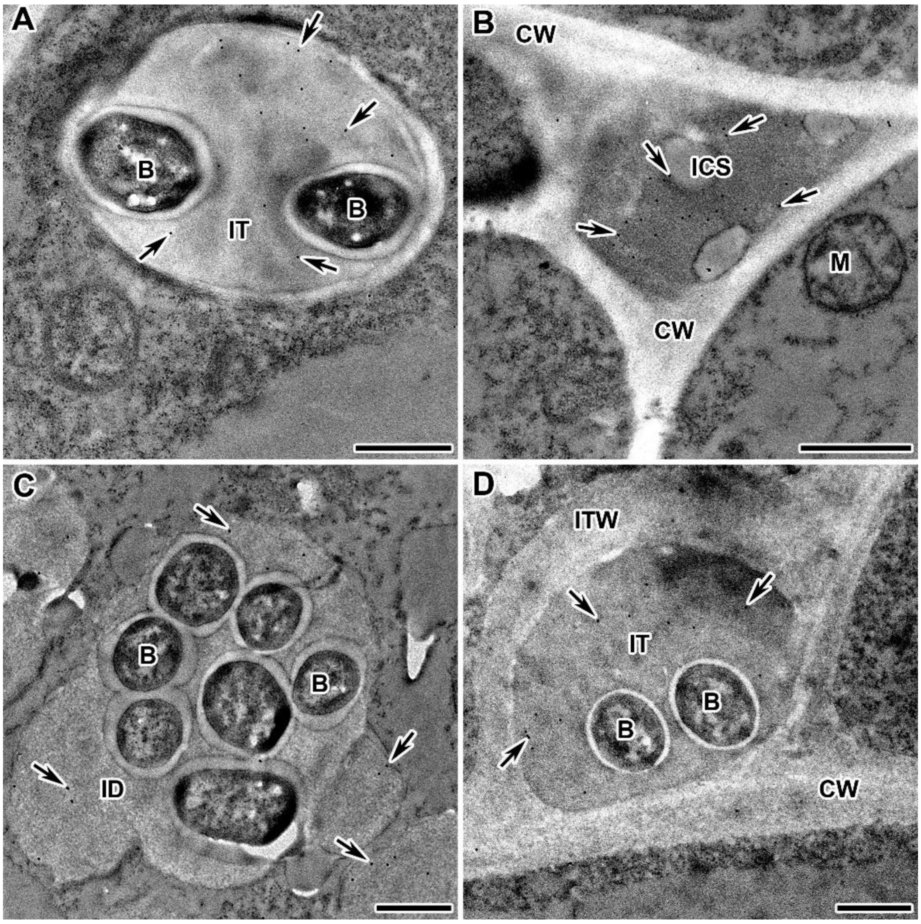

Figure S3. Immunogold localisation of extensin labelled with JIM11 in nodules from wild-type and mutant *P. sativum*. The secondary antibody used was the goat anti-rat IgG monoclonal antibody conjugated to 10 nm diameter colloidal gold. (**A**) Infection thread in the wild-type SGE; (**B**) three-cell junction in the wild-type SGE; (**C**) infection droplet in the mutant SGEFix⁻-1 (*sym40-1*); (**D**) infection thread in the mutant SGEFix⁻-2 (*sym33-3*). CW—cell wall, M—mitochondrion, ICS—intercellular space, IT—infection thread, ITW—infection thread wall, ID—infection droplet, B—bacterium; arrows indicate gold particles. Bar = 500 nm.

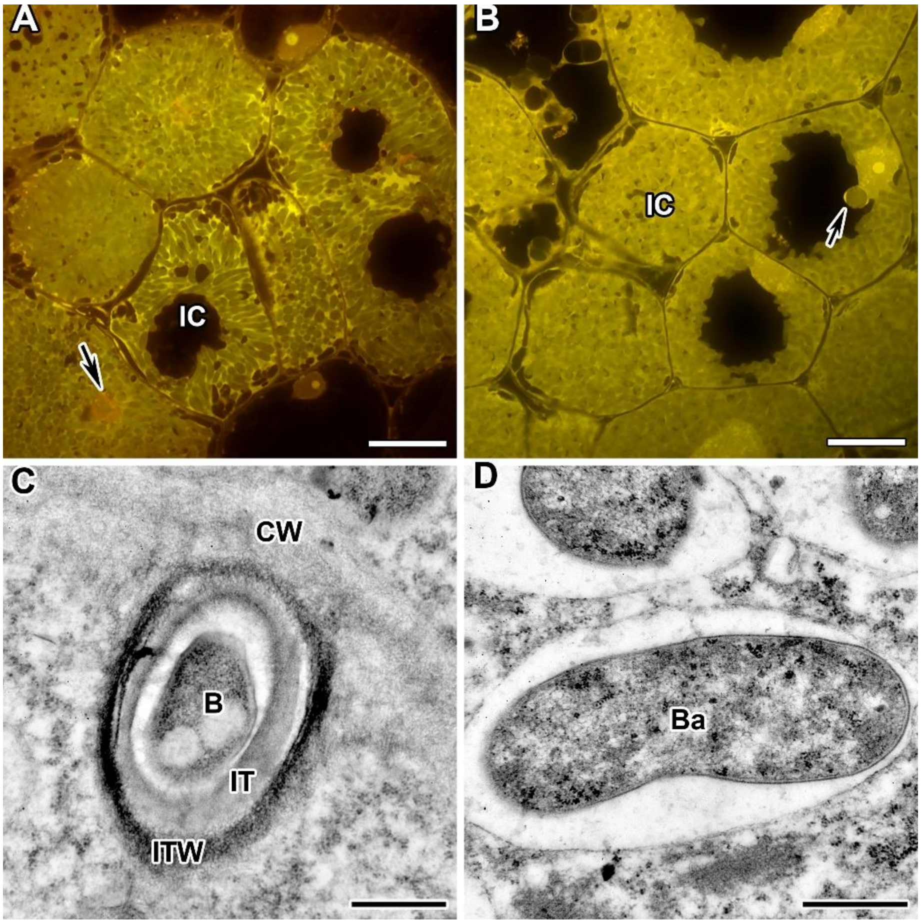

Figure S4. Fluorescent and transmission electron micrographs of cells from *P. sativum* (**B-D**) and *M. truncatula* (**A**) nodules treated as a negative control. (**A**) Wild-type *M. truncatula* A17 labelled with LM20 and treated with the anti-mouse secondary antibody conjugated with Alexa Fluor 488. (**B**) Wild-type *P. sativum* SGE after the omission of the primary antibody. (**C**) Wild-type *P. sativum* SGE labelled with CCRC-M1 and treated with the anti-rat secondary antibody conjugated to 10 nm diameter colloidal gold. (**D**) Wild-type *P. sativum* SGE after the omission of the primary antibody. Fluorescent labelling was absent when cells were treated with the unspecific secondary antibody (**A**), after the omission of the primary antibody (**B**). Gold particles were absent when cells were treated with the unspecific secondary antibody (**C**), after the omission of the primary antibody (**D**). IC—infected cell, IT—infection thread, ITW—infection thread wall, B—bacterium, Ba—bacteroid, arrowheads indicate infection thread. Bars (**A,B**) = 20 μm, (**C**) = 500 nm, (**D**) = 200 nm.
